# Supplementary material for: Impact of Partnered Pharmacist Medication Charting (PPMC) on Medication Discrepancies and Errors: A Pragmatic Evaluation of an Emergency Department-Based Process Redesign
Source: Int J Environ Res Public Health. 2023 Jan 13;20(2):1452. doi: 10.3390/ijerph20021452 (PMC9859430; doi:10.3390/ijerph20021452)
Supplement: Supplementary file 1 [file ijerph-20-01452-s001.zip › ijerph-2131884-supplementary.pdf]

Electronic supplementary material

Supplementary Tables

Supplementary Table S1. List of high-risk medicines.

Class	Medicines
**Antimicrobials**	**Aminoglycosides:** Gentamicin, tobramycin, amikacin, netilmicin<br>Vancomycin, amphotericin
**Potassium and other electrolytes**	Injections of concentrated electrolytes: potassium, magnesium, calcium, hypertonic sodium chloride.
**Insulin**	All insulins
**Narcotics or opioids and other sedatives**	**Opioids:** Alfentanil, buprenorphine, codeine, fentanyl, hydromorphone. Methadone, morphine, oxycodone, pethidine, remifentanil, tapentadol, tramadol
	**Benzodiazepines:** Alprazolam, bromazepam, clobazam, clonazepam, diazepam, flunitrazepam, lorazepam, midazolam, nitrazepam, oxazepam, temazepam
	**Analgesic patches:** E.g., Buprenorphine
	**Short-term anaesthetics:** Thiopentone, propofol, ketamine
**Heparin and other anticoagulants**	**Vitamin K antagonists:** Warfarin
	**Heparins:** Heparin
	**LMWH:** Enoxaparin, dalteparin, nadroparin, danaparoid
	**Direct thrombin inhibitors:** Bivalirudin, dabigatran
	**Factor Xa inhibitors:** Apixaban, rivaroxaban, fondaparinux
**Chemotherapeutic agents**	**Nitrogen mustards:** Bendamustine, chlorambucil, cyclophosphamide, ifosfamide, melphalan
	**Nitrosoureas:** Carmustine, fotemustine, lomustine
	**Triazenes:** Dacarbazine, temozolomide
	**Other alkylating agents:** Busulfan, procarbazine
	**Anthracyclines:** Daunorubicin, doxorubicin, epirubicin, idarubicin, mitozantrone
	**Folic acid antagonists:** Methotrexate, pemetrexed, pralatrexate, raltitrexed
	**Purine antagonists:** Cladribine, clofarabine, fludarabine, mercaptopurine, tioguanine
	**Pyrimidine antagonists:** Azacitidine, capecitabine, cytarabine, fluorouracil, gemcitabine, trifluridine with tipiracil
	**Other antimetabolites:** Hydroxycarbamide
	**Platinum compounds:** Carboplatin, cisplatin, oxaliplatin
	**Proteasome inhibitors:** Bortezomib, carfilzomib
	**Taxanes:** Cabazitaxel, docetaxel, paclitaxel
	**Topoisomerase I inhibitors:** Irinotecan, topotecan
	**Vinca alkaloids:** Vinblastine, vincristine, vinorelbine
	**Other cytotoxic antineoplastics:** Bleomycin, brentuximab vedotin, dactinomycin, eribulin, etoposide, gemtuzumab ozogamicin, inotuzumab ozogamicin, mitomycin, niraparib, olaparib, romidepsin, trabectedin, trastuzumab, emtansine, venetoclax, vorinostat
**Drugs with narrow therapeutic index***	**Antiarrhythmics**: Amiodarone, digoxin, flecainide, lignocaine, quinidine
	**Antiepileptics**: Carbamazepine, phenytoin, phenobarbitone, sodium valproate
	**Mental health medicines**: Lithium, clozapine
	**Others:** Theophylline, levothyroxine, perhexiline, salicylate, mexiletine

*In addition to the National Safety and Quality Health Service (NSQHS) list of APINCH high-risk medicines, drugs with a narrow therapeutic index were also included. The list was developed using the Australian Medicines Handbook online accessed via the University of Tasmania.

Supplementary Table S2. List of Tasmanian Health Service (THS) time-critical medicines.

Time Critical Medicines

A missed dose of a time critical medicine could have serious negative outcomes for a patient. It is imperative that doses of these medicines are not missed or vastly delayed.

	Some examples	Possible outcomes if dose is missed
Anticoagulants	Heparin, warfarin, apixaban	Venous thromboembolism (DVT, PE); stroke
Anticonvulsants	Carbamazepine, phenytoin	Increased seizure activity, especially if dose is missed peri-operatively
Antidotes (usually STAT orders)	Naloxone, calcium folinate	Toxicity or harm
IV Antimicrobials	IV antibiotics and antifungals	Sepsis, worsening or prolonged infection, resistance
Hypoglycaemic agents	Insulin, oral hypoglycaemics	Ketoacidosis, hyperglycaemia
Antiparkinsons medications	Levodopa combinations	Reduction of function, slow recovery
Corticosteroids and hormones	Cortisone, desmopressin, prednisolone	Hypotensive crisis, delayed symptom control, worsening of condition
Immunosuppressants	Ciclosporin, tacrolimus	Rejection of transplants
Cytotoxics	Methotrexate, cyclophosphamide	Treatment failure or incomplete remission
Clozapine	Clozapine	Re-titration of clozapine dose, recurrence of symptoms

TASMANIAN HEALTH SERVICE
Tasmanian Government

Using the above THS TCM categories as a reference, the following lists were developed using the online Australian Medicines Handbook, which was accessed via the University of Tasmania. Medicines with unspecified indications in the patient's medical records were excluded.

Drug class	Medicines
**Anticoagulants**	**Vitamin K antagonists:** Warfarin
	**Heparins:** Heparin
	**LMWH:** Enoxaparin, dalteparin, nadroparin, danaparoid
	**Direct thrombin inhibitors:** Bivalirudin, dabigatran
	**Factor Xa inhibitors:** Apixaban, rivaroxaban, fondaparinux
**Anticonvulsants**	**Barbiturates:** Phenobarbital, primidone
	**Benzodiazepines:** Clobazam, clonazepam, diazepam, midazolam
	**Other antiepileptics:** Acetazolamide, brivaracetam, carbamazepine, ethosuximide, gabapentin, lacosamide, lamotrigine, levetiracetam, oxcarbazepine, perampanel, phenytoin, pregabalin, rufinamide, stiripentol, sulthiame, tiagabine, topiramate, valproate, vigabatrin, zonisamide
**Antidotes (usually STAT order)**	Naloxone, protamine, resonium, folinic acid, calcium folinate
**IV antimicrobials**	IV antibiotics and IV antifungals
**Hypoglycaemic agents**	Insulins
	**Sulfonylureas:** Glibenclamide, gliclazide, glimepiride, glipizide
	**Dipeptidyl peptidase-4 inhibitors:** Alogliptin, alogliptin with metformin, linagliptin, linagliptin with metformin, saxagliptin, saxagliptin with dapagliflozin, saxagliptin with metformin, sitagliptin, sitagliptin with metformin, vildagliptin, vildagliptin with metformin

	**Sodium-glucose co-transporter 2 inhibitors:** Dapagliflozin, dapagliflozin with metformin, empagliflozin, empagliflozin with linagliptin, empagliflozin with metformin, ertugliflozin, ertugliflozin with metformin, ertugliflozin with sitagliptin
	**Other drugs for diabetes:** Acarbose, insulin, metformin, metformin with glibenclamide, pioglitazone
**Antiparkinson medications**	**Dopamine agonists:** Apomorphine, pramipexole, ropinirole, rotigotine
	**Ergot derivatives:** Bromocriptine, cabergoline
	**Anticholinergics:** Atropine, benzatropine, darifenacin, hyoscine hydrobromide, orphenadrine, oxybutynin, solifenacin, tolterodine, trihexyphenidyl, glycopyrronium, hyoscine, propantheline
	**Monoamine oxidase type B inhibitors:** Rasagiline, safinamide, selegiline
	**Other drugs for Parkinson's disease:** Amantadine, entacapone, levodopa
**Corticosteroids and hormones (PO or IV)**	**Corticosteroids:** Desmopressin, betamethasone, cortisone, dexamethasone, hydrocortisone, methylprednisolone, prednisolone, prednisone, triamcinolone
	**Antidiuretic hormone agonists and antagonists:** Argipressin, demeclocycline, ornipressin, terlipressin, tolvaptan
**Immunosuppressants**	**Calcineurin inhibitors:** Ciclosporin, cyclosporin, tacrolimus
**Cytotoxic agents**	**Nitrogen mustards:** Bendamustine, chlorambucil, cyclophosphamide, ifosfamide, melphalan
	**Nitrosoureas:** Carmustine, fotemustine, lomustine
	**Triazenes:** Dacarbazine, temozolomide
	**Other alkylating agents:** Busulfan, procarbazine
	**Anthracyclines:** Daunorubicin, doxorubicin, epirubicin, idarubicin, mitozantrone
	**Folic acid antagonists:** Methotrexate, pemetrexed, pralatrexate, raltitrexed
	**Purine antagonists:** Cladribine, clofarabine, fludarabine, mercaptopurine, tioguanine
	**Pyrimidine antagonists:** Azacitidine, capecitabine, cytarabine, fluorouracil, gemcitabine, trifluridine with tipiracil
	**Other antimetabolites:** Hydroxycarbamide
	**Platinum compounds:** Carboplatin, cisplatin, oxaliplatin
	**Proteasome inhibitors:** Bortezomib, carfilzomib
	**Taxanes:** Cabazitaxel, docetaxel, paclitaxel
	**Topoisomerase I inhibitors:** Irinotecan, topotecan
	**Vinca alkaloids:** Vinblastine, vincristine, vinorelbine
	**Other cytotoxic antineoplastics:** Bleomycin, brentuximab vedotin, dactinomycin, eribulin, etoposide, gemtuzumab ozogamicin, inotuzumab ozogamicin, mitomycin, niraparib, olaparib, romidepsin, trabectedin, trastuzumab, emtansine, venetoclax, vorinostat
**Psychotropic medication**	Clozapine

Supplementary Table S3. Definitions and examples of each medication discrepancy or error.

Types	Definitions*
Omitted drug[†]	A drug in a patient's medication history (BPMH) is not charted on the medication chart without a reason for the omission documented.<br><br>**Undocumented likely intentional discrepancy**: A patient admitted normally takes 100mg metoprolol BD for rate control of their AF. On admission, they are hemodynamically unstable (hypotensive and bradycardic). The metoprolol is not charted and the rationale is not documented, but it is clinically appropriate to withhold the medication.<br><br>**Unintentional error**: A patient admitted normally takes 100mg metoprolol BD for rate control of their AF. On admission, they are hemodynamically stable, but their metoprolol is not charted. The medication is recommenced post-medication reconciliation (MedRec) or at discharge.
Different but similar drug	A different drug was charted than the usual drug taken by the patient prior to admission but was classified in the same Anatomical Therapeutic Chemical (ATC) Classification System Level 3 without a reason for the change documented.<br><br>**Undocumented likely intentional discrepancy**: A patient regularly takes pantoprazole 20mg daily for GORD. They are admitted for gastritis and changed to esomeprazole 40mg daily to allow for IV dosing. The change is not documented but is clinically appropriate.<br><br>**Unintentional error**: A patient normally takes OxyContin 10mg BD prior to admission. They are charted for MS Contin 10mg BD. The medication is reverted to the pre-admission medication post-MedRec or at discharge.
Added drug	A drug was added to a patient's regimen without a reason for the change documented.<br><br>**Undocumented likely intentional discrepancy**: patient's furosemide was ceased 2 weeks prior to admission for dehydration. The patient is admitted with fluid overload and furosemide is recommenced. Although the rationale is not documented, the change is clinically appropriate.<br><br>**Unintentional error**: A patient ceased lamotrigine 2 weeks prior to admission due to the development of a rash. Lamotrigine was unintentionally charted on admission. The medication is ceased post-MedRec or at discharge.
Different dose	A dose written on the chart differed from the usual dose taken by the patient prior to admission without a reason for the change documented.<br><br>**Undocumented likely intentional discrepancy**: A patient admitted following an alcohol binge has esomeprazole changed from 20mg daily to 40mg daily. The change is not documented but is clinically appropriate.<br><br>**Unintentional error**: A patient admitted with AKI takes pregabalin 75mg BD prior to hospitalisation. On admission, the patient is charted for 150mg BD pregabalin. The medication is reverted to the pre-admission medication dose post-MedRec or at discharge.
Different frequency	A frequency written on the chart differed from the frequency used by the patient prior to admission without a reason for the change documented.<br><br>**Undocumented likely intentional discrepancy**: Prior to admission, a patient takes paracetamol 1g QID regularly. The patient is charted for 1g TDS. It is noted that the patient weighs 50kg, so the frequency change is clinically appropriate, but the rationale is not documented.<br><br>**Unintentional error**: A patient was admitted to the hospital for appendicitis. His olanzapine is charted as 10mg daily. He was regularly on 10mg BD prior to admission.

	The medication is reverted to the pre-admission medication frequency post-MedRec or at discharge.
Different route	A route written on the chart differed from the route used by the patient prior to admission without a reason for the change documented.<br><br>**Undocumented likely intentional discrepancy:** A patient takes flucloxacillin 500 mg QID (oral) prior to admission for a diabetic foot infection. On admission, the patient was commenced on flucloxacillin 1g QID (IV) due to deterioration of the foot. The change is not documented but is clinically appropriate.<br><br>**Unintentional error**: A patient is taking intranasal desmopressin prior to hospitalisation. On admission, the patient is charted for oral desmopressin. The medication is reverted to the pre-admission medication route post-MedRec or at discharge.
Different dosage form	A dosage form written on the chart differed from the usual dosage form used by the patient prior to admission without a reason for the change documented.<br><br>**Undocumented likely intentional discrepancy**: a patient was charted for a paracetamol 1g QID following admission for appendicitis with acute pain. Prior to admission, the patient was taking paracetamol SR 1330mg TDS. The change is undocumented but clinically appropriate.<br><br>**Unintentional error**: a patient was charted for a non-sustained release formulation of quetiapine when taking the sustained-release form prior to admission. The medication is reverted to the pre-admission dosage form post-MedRec or at discharge.
Incomplete order	Omission of a pre-admission medication's dose, frequency or both in the appropriate sections of the medication chart.<br><br>**Undocumented likely intentional discrepancy**: A patient's own alternative medicine is charted. A strength is not documented as the patient manages this medication themselves.<br><br>**Unintentional error**: a strength of metformin was not written in the chart.

*All regular medications, including medically indicated complementary alternative medicines, and medically prescribed as-needed (PRN) medications for the management of episodic acute conditions or acute exacerbations of chronic conditions are included in these definitions. Examples of PRN medications include glyceryl trinitrate sublingual for acute episodes of angina, salbutamol/other relievers for asthma/COPD, triptans for acute migraine attacks, opioid analgesics PRN for breakthrough pain in chronic pain, quetiapine/other antipsychotics PRN for agitation and other psychotropic PRN medications. Examples of medically indicated complementary alternative medicines include vitamin D for osteopenia and multivitamin for malnutrition or chronic alcohol abuse.

†The study also examined whether the appropriate drug chart (e.g., insulin chart) was used, as a secondary outcome.

Supplementary Table S4. Occurrence of undocumented medication discrepancies.

Discrepancies	Study group			*P*-value	
	**PPMC** (N = 230 patients)	**Early BPMH** (N = 230 patients)	**Usual care** (N = 588 patients)	**Overall**	**Pairwise**
Total discrepancies	22	360	1042		
Discrepancies, median (IQR)	0 (0, 0)	1 (0, 2)	1 (1, 3)	< 0.001*	< 0.001[†‡], 0.023[§]
Discrepancies per initially charted medicines, median (IQR)	0 (0, 0)	0.1 (0, 0.2)	0.1 (0, 0.3)	< 0.001*	< 0.001[†‡], 0.005[§]
Patients with ≥ 1 discrepancy, n (%)	14 (6.1%)	137 (59.6%)	409 (69.6%)	< 0.001[¶]	
Patients with				< 0.001[¶]	
0 discrepancy	216 (93.9%)	93 (40.4%)	179 (30.4%)		
1 discrepancy	9 (3.9%)	50 (21.7%)	155 (26.4%)		
2 discrepancies	2 (0.9%)	35 (15.2%)	97 (16.5%)		
3 discrepancies	3 (1.3%)	20 (8.7%)	56 (9.5%)		
4 discrepancies	0 (0%)	10 (4.3%)	44 (7.5%)		
≥ 5 discrepancies	0 (0%)	22 (9.6%)	57 (9.7%)		
**Discrepancies involving medicines**					
Regular medicines	15 (68.2%)	312 (86.7%)	879 (84.4%)		
Medically prescribed PRN medicines	3 (13.6%)	23 (6.4%)	75 (7.2%)		
Medically indicated complementary medicines	4 (18.2%)	25 (6.9%)	88 (8.4%)		

Abbreviations: BPMH, best-possible medication history; n, number; PPMC, partnered pharmacist medication charting.
*Kruskal-Wallis rank sum test with Dunn's post-hoc test: [†]PPMC *vs* early BPMH; [‡]PPMC *vs* usual care; [§]early BPMH *vs* usual care.
[¶]Pearson's Chi-squared test

Supplementary Table S5. Estimating the extrapolated prevalence and clinical severity of errors from the panel assessment findings.

Outcome	Measure	PPMC	Early BPMH	Usual care
	**[A]** Patients	230	230	588
	**[B]** Charted medications in initial National Inpatient Medication Chart			
	**[B$_1$]** Total charted medications	2515	2277	5328
	**[B$_2$]** Total charted HRMs	416	393	800
	**[B$_3$]** Total charted TCMs	433	405	725
Discrepancies	**[C]** Total discrepancies identified, n	22	360	1042
	**[C$_1$]** Patients with $\geq$ 1 discrepancy, n (% of the patients)	14 (6.1%)	137 (59.6%)	409 (69.6%)
	**[C$_2$]** Discrepancies per 100 prescribed medications = **[C]/[B$_1$]*100**	0.9	15.8	19.6
	**[C$_3$]** Discrepancies involving HRMs, n (% of charted HRMs)	5 (1.2%): 5/416	58 (14.8%): 58/393	132 (16.5%): 132/800
	**[C$_4$]** Discrepancies involving TCMs, n (% of charted TCMs)	1 (0.2%): 1/433	34 (8.4%): 34/405	71 (9.8%): 71/725
Panel assessment findings	**[D]** Assessed discrepancies	All 22 discrepancies (in 230 patients)	54 randomly selected discrepancies (in 41 patients)	82 randomly selected discrepancies (in 68 patients)
	**[E]** Discrepancies deemed unintentional medication errors, %	63.6%: 14 errors/22 discrepancies *100	87.0%: 47 errors/54 discrepancies * 100	87.8%: 72 errors/82 discrepancies * 100
	**[E$_1$]** Low risk, number of errors (%)	9 (64.3%)	7 (14.9%)	20 (27.8%)
	**[E$_2$]** Moderate risk, number of errors (%)	2 (14.3%)	20 (42.6%)	24 (33.3%)
	**[E$_3$]** High risk, number of errors (%)	3 (21.4%)	17 (36.2%)	25 (34.7%)
	**[E$_4$]** Extreme risk, number of errors (%)	0 (0%)	3 (6.4%)	3 (4.2%)
	**[F]** Patients with $\geq$ 1 error, %	8 of 230 patients = 3.5%	34 of 41 patients = 82.9%	60 of 68 patients = 88.2%
	**[G]** Patients with $\geq$ 1 error bearing high or extreme risk, %	3 of 230 patients = 1.3%	16 of 41 patients = 39.0%	26 of 68 patients = 38.2%
	**[H]** Errors involving HRMs, n (% of assessed discrepancies involving HRMs)	1 (20%): 1 errors/5 discrepancies	14 (100%): 14 errors/14 discrepancies	15 (93.8%): 15 errors/16 discrepancies
	**[I]** High/extreme risk errors involving HRMs, n (% of errors involving HRMs)	0 (0%): 0 high/extreme risk errors/1 error)	7 (50%): 7 high/extreme risk errors/14 errors	10 (66.7%); 7 high/extreme risk errors/15 errors
	**[J]** Errors involving TCMs, n (% of assessed discrepancies involving TCMs)	1 (100%): 1 errors/1 discrepancies	6 (85.7%): 6 errors/7 discrepancies	11 (100%): 11 errors/11 discrepancies
	[**K**] High/extreme risk errors involving TCMs, n (% of errors involving TCMs)	0 (0%): 0 high/extreme risk errors of the 1 error	4 (66.7%); 4 high/extreme risk errors of the 6 errors	4 (36.4%): 4 high/extreme risk errors of the 11 errors
Projected findings	**[L]** Total unintentional medication errors: n, % (95% CI) = **[C] * [E]**	14 (63.6%: 14/22)	313 (87.0% * 360)	915 (87.8% * 1042)
	**[L$_1$]** Low risk, number of errors: n, % (95% CI) = **[L] * [E$_1$]**	9, 40.9% (20.4% to 61.5%)	47, 14.9% (11.0% to 18.8%)	254, 27.8% (95%CI: 24.9–30.7)
	**[L$_2$]** Moderate risk, number of errors: n, % (95% CI) = **[L] * [E$_2$]**	2, 9.1% (0% to 21.1%)	133, 42.6% (37.1% to 48.0%)	305, 33.3% (30.3% to 36.4%)
	**[L$_3$]** High risk, number of errors: n, % (95% CI) = **[L] * [E$_3$]**	3, 13.6% (0% to 28.0%)	113, 36.2% (30.8% to 41.5%)	318, 34.7% (31.6% to 37.8%)
	**[L$_4$]** Extreme risk, number of errors: n, % (95% CI) = **[L] * [E$_4$]**	0, 0% (0% to 0%)	20, 6.4% (3.7% to 9.1%)	38, 4.2% (2.9% to 5.5%)
	**[M]** Patients with $\geq$ 1 error, % (95% CI) = **[C$_1$] * [F]**	3.5% (1.1% to 5.8%)	49.4% (42.5% to 56.3%)	61.4% (56.3% to 66.7%)
	**[N]** Patients with $\geqslant$ 1 high/extreme risk error: % (95% CI)=**[C$_1$] * [G]**	1.3% (0% to 2.8%)	23.2% (14.4% to 32.1%)	26.6% (18.6% to 34.6%)
	Errors per 100 prescribed medications, ratio (95% CI): **[E]/[B$_1$] * 100**	0.6 (0.3 to 0.8)	13.8 (12.3 to 15.2)	17.2 (16.2 to 18.2)
	Errors bearing high/extreme risk per 100 prescribed medications, ratio (95% CI): **[E$_3$ + E$_4$]/[B$_1$] * 100**	0.1 (0 to 0.3)	5.9 (4.9 to 6.8)	6.7 (6.0 to 7.3)

	Errors involving HRMs: n (**[C_3] * [H]**), % of charted HRMs (95% CI)	n=1, 0.2% (0% to 0.7%)	n~58, 14.8% (11.3% to 18.3%): 58/393	n~124, 15.5% (13% to 18%)
	High/extreme risk errors involving HRMs: n ([**C_3**] * [I**]**), % of charted HRMs (95% CI)	n=0, 0% (0% to 0%)	n~29, 7.4% (4.8% to 10%)	n~88, 11% (8.9% to 13.2%)
	Errors involving TCMs: n ([**C_4**] * [J]), % of charted TCMs (95% CI)	n=1, 0.2% (0% to 0.7%)	n~29, 7.2% (4.7% to 9.7%)	n~71, 9.8% (7.6% to 12%)
	High/extreme risk errors involving TCMs: n ([**C_4**] * [K**]**), % of charted TCMs (95% CI)	n=0, 0% (0% to 0%)	n~19, 4.8% (2.7% to 6.9%)	n~26, 3.6% (2.2% to 4.9%)

Abbreviations: BPMH, best-possible medication history; CI, confidence interval; HRMs, high-risk medicines; n, number; PPMC, partnered pharmacist medication charting; TCMs, time-critical medicines

Supplementary Table S6. Case vignettes for errors bearing high or extreme risk.

**Regular regimen**	**Error type**	**MedRec note**	**Case description**	**Possible consequence**	**Potential risk**
Subcutaneous insulin (Ryzodeg 70/30) twelve units twice each day	Omitted drug	"*Missing – undocumented. patient's regular insulin [is] NOT charted ATOR [at time of review], please review asap [as soon as possible] and chart [it] if appropriate to continue.*"	A patient with type-2 diabetes presented to the hospital following two witnessed syncopal events, which were thought to be secondary to antihypertensives.	Hyperglycaemia	Extreme risk
Insulin glargine (Optisulin) 28 units subcutaneously at bedtime	Omitted drug	"*Missing - undocumented.* "*Please chart pt's regular insulin (Optisulin).*"	A patient, in their mid-80s, presented with three days of shortness of breath and a worsening productive cough. Blood glucose was 5.3 mmol/L during the admission	Hyperglycaemia	Extreme risk
Quetiapine in the morning (50 mg), mid-day (50 mg) and evening (100 mg)	Different dose/frequency	"*Change – undocumented: As per med hx [history] patient has regular morning and midi doses of quetiapine in addition to the nocte dose.*"	A patient, in their early 60s, presented with symptoms of alcohol withdrawal in the context of binge drinking on the day before admission. The patient also voiced feelings of being unsafe at home due to intermittent thoughts of suicide. The patient had been in a low mood with suicidal ideation and anhedonia for months.	Worsening psychiatric symptoms	High risk
Dabigatran 110mg one capsule twice each day	Different dose	"*Changed – Undocumented: "[X] takes 110mg twice a day at home, currently prescribed 150mg twice a day as [an] inpatient. Given [X]'s eGFR is 46 and [X] is 87 years old, the recommended maximum dose is 110mg twice a day. Please rechart as 110mg twice a day.*"	A patient, in their mid-80s, presented with an infection of the right index finger. The patient was diagnosed with right-hand cellulitis and initiated on IV flucloxacillin.	Haemorrhage	High risk
Apixaban 2.5mg one tablet twice each day	Omitted drug	"*Missing – Undocumented. Regular medication missed. Please chart if appropriate.*"	A 73-year-old male patient presented to the RHH ED with diaphoresis, headache and being unable to self-measure his blood pressure (BP). The BP was 240/100 mmHg at the highest on presentation.	Stroke	Extreme risk
Perindopril/indapamide (4mg + 1.25mg) one tablet each morning for hypertension.	Omitted drug	"*Missing – Undocumented.: Pre-admission medication not charted. Recommendation: Team to review and consider charting antihypertensive, note that [the] patient's BP [blood pressure] hasn't been stable.*"	A patient, in their mid-50s, presented with undifferentiated epigastric pain. On examination, oxygen saturation was 92% (on room air) and the patient was afebrile. Other observations were within the normal range. Minimal peripheral oedema was observed. The patient was subsequently diagnosed with remnant cholecystitis, on the background of having a laparoscopic cholecystectomy in 2019 and was conservatively managed with IV antibiotics and supportive care in the ward.	Hypertensive crisis	High risk

Abbreviations: MedRec, medication reconciliation; mg, milligram

Supplementary Figures

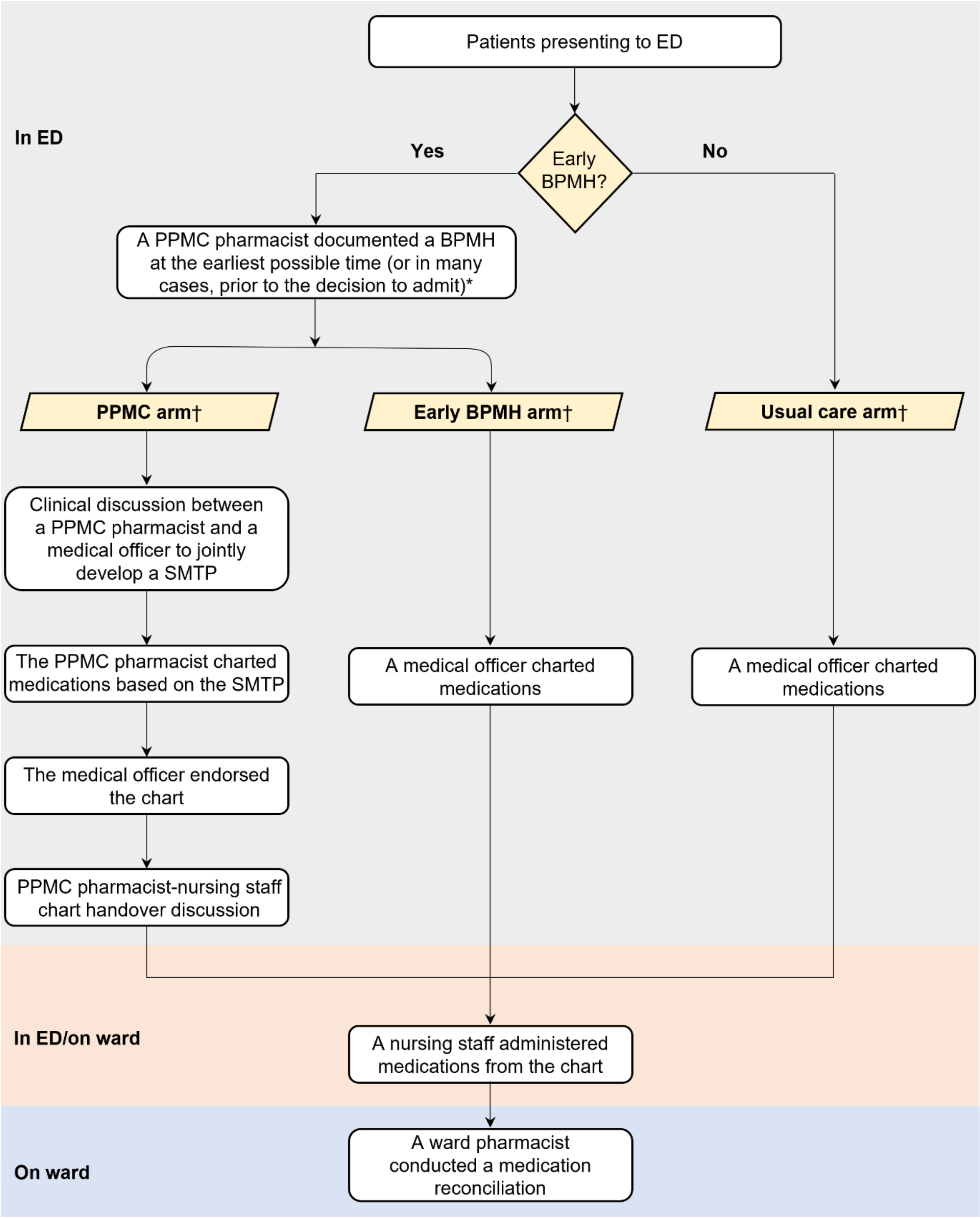

Supplementary Figure S1. Admission processes for patients in the PPMC, early BPMH and usual care arms.

BPMH, best-possible medication history; ED, emergency department; PPMC, partnered pharmacist medication charting; SMTP, shared medication treatment plan

*Alternatively, the BPMH might have been collected by non-credentialled pharmacists and subsequently provided to the PPMC pharmacist.

†ED staff allocated patients to either of the study arms as part of their routine medical care.

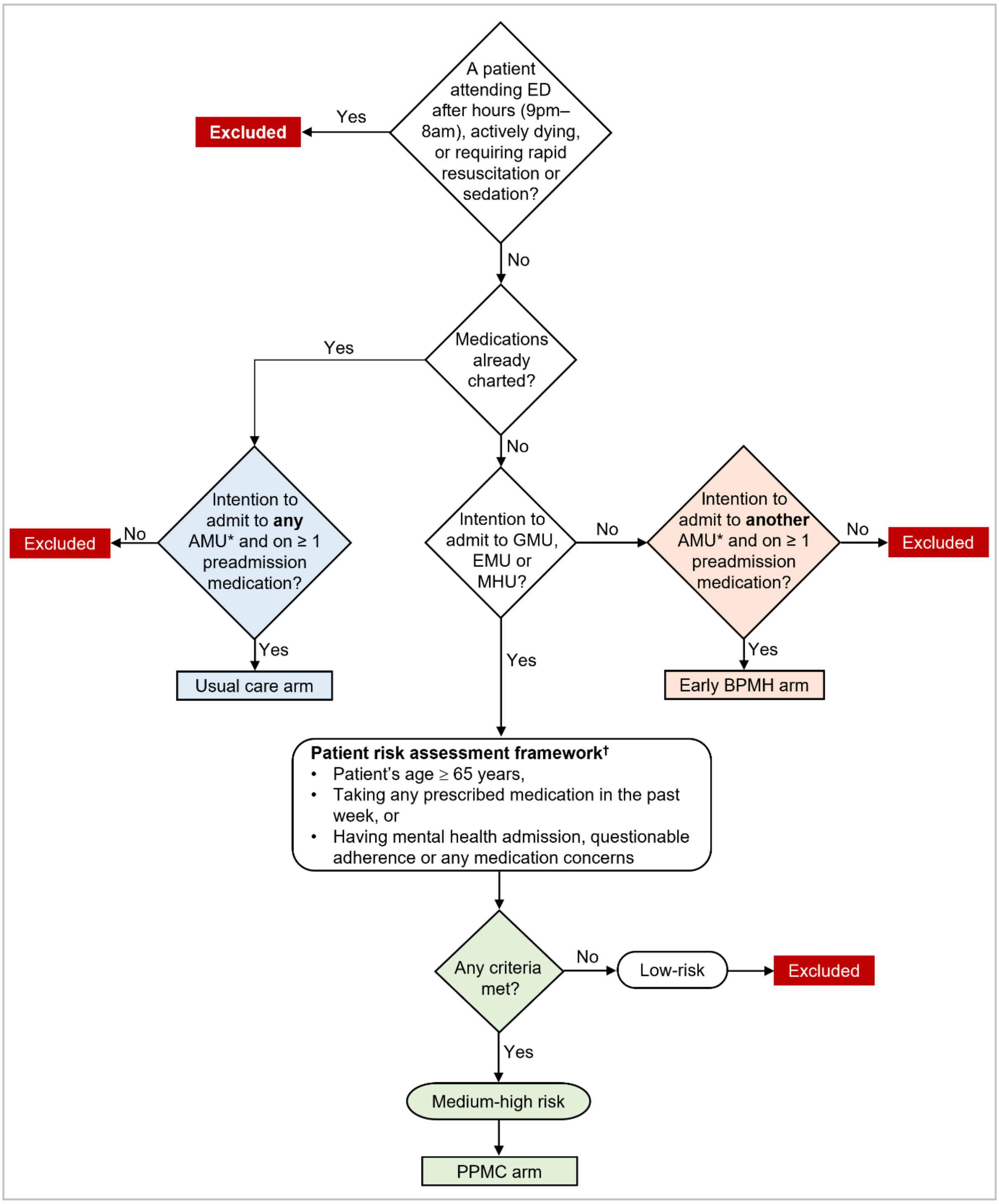

Supplementary Figure S2. Screening and assignment of patients into study arms.

Abbreviations: AMU, acute medical units; ED, emergency department; EMU, emergency medicine unit; GMU, general medicine unit; MHU, mental health unit; PPMC, partnered pharmacist medication charting.

*Patients admitted primarily for medical reasons receive care in AMUs (acute medical units), which consisted of a wide range of medical specialities such as general medicine, emergency medicine, mental health, cardiology, respiratory, renal and stroke units.

†Developed by the PPMC working group

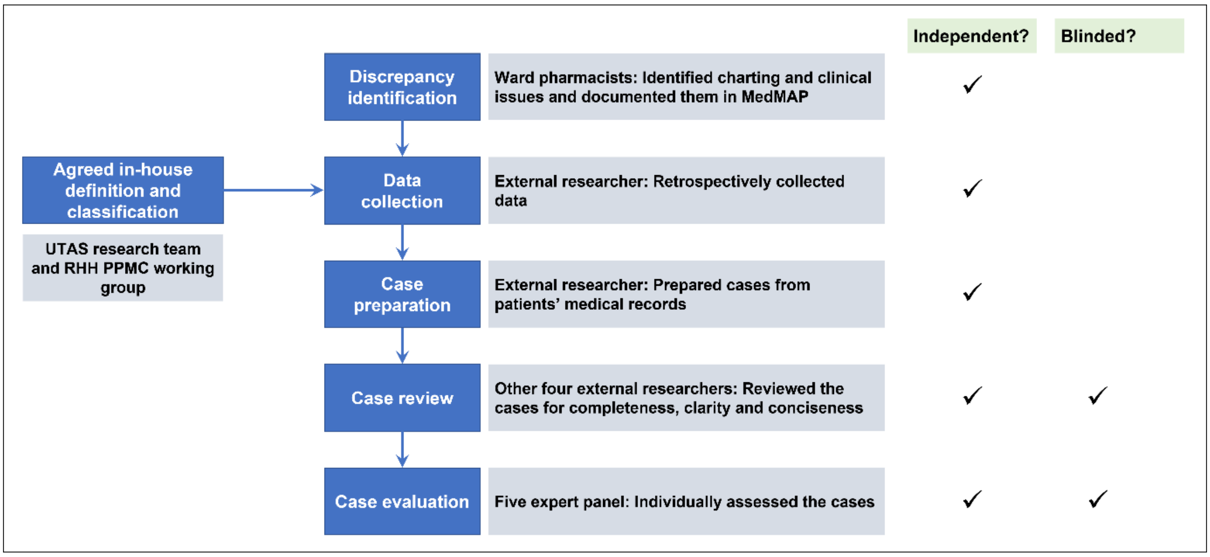

Supplementary Figure S3. Assessment process flowchart illustrating steps in identification, collection, preparation, review and evaluation of discrepancies/errors.

Abbreviations: MedMAP, medication management action plan; PPMC, partnered pharmacist medication charting; RHH, Royal Hobart Hospital; UTAS, University of Tasmania.
